# Deletion of a xenobiotic metabolizing gene in mice affects folate metabolism

**DOI:** 10.1016/j.bbrc.2007.10.026

**Published:** 2007-12-21

**Authors:** Larissa Wakefield, Valerie Cornish, Hilary Long, William J. Griffiths, Edith Sim

**Affiliations:** a Department of Pharmacology, University of Oxford, Mansfield Road, Oxford OX1 3QT, UK; b The Department of Pharmaceutical & Biological Chemistry, The School of Pharmacy, University of London, 29/39 Brunswick Square, Bloomsbury, London WC1N 1AX, UK; c Institute of Mass Spectrometry, School of Medicine, University of Wales Swansea, Singleton Park, Swansea SA2 8PP, UK

**Keywords:** NAT/arylamine *N*-acetyltransferase, Folate, NTD, Gender, Sex ratio, Breast cancer

## Abstract

The mouse arylamine *N*-acetyltransferase 2 (Nat2) and its homologue (NAT1) in humans are known to detoxify xenobiotic arylamines and are also thought to play a role in endogenous metabolism. Human *NAT1* is highly over-expressed in estrogen receptor positive breast tumours and is implicated in susceptibility to neural tube defects. *In vitro* assays have suggested an endogenous role for human NAT1 in folate metabolism, but *in vivo* evidence to support this hypothesis has been lacking. Mouse Nat2 provides a good model to study human NAT1 as it shows similar expression profiles and substrate specificities. We have generated transgenic mice lacking a functional *Nat2* gene and compared the urinary levels of acetylated folate metabolite *para*-aminobenzoylglutamate in *Nat2* knockout and *Nat2* wild-type mice. These results support an *in vivo* role for mouse Nat2/human NAT1 in folate metabolism. In addition, effects of the *Nat2* deletion on sex ratios and neural tube development are described.

Arylamine *N*-acetyltransferases have well-defined roles in detoxification of xenobiotic arylamines and hydrazines. There are two *NAT* genes in humans, showing distinct substrate specificities [1] and expression profiles [2], with human *NAT2* expressed mainly in the liver and intestine, and widely held to have a role in xenobiotic metabolism. Human *NAT1* and its homologue in mouse (mouse *Nat2*) is strongly expressed in the placenta, early in embryonic development [3] in the developing neural tube [4] and in folate-sensitive cells contributing to the neuroendocrine system [5], as well as in the adult, in erythrocytes and a range of epithelia [2,6].

Both human *NAT1* and human *NAT2* genes are polymorphic and there are suggestions that the polymorphic status may influence susceptibility to cancers in epithelia exposed to xenobiotic toxins [7]. Recent studies linking levels of *NAT1* expression with those of the estrogen receptor in breast tumour tissue [8] indicate that the role of NAT1 in carcinogenesis and tumour growth in some tissues relates to endogenous as opposed to xenobiotic metabolism. *In vitro* studies have shown that human NAT1 is able to acetylate the folate catabolite *para*-aminobenzoylglutamate (*p*ABAglu) [9,10], however, to date, there has been no confirmation of this activity *in vivo*. We have used a well-characterized transgenic mouse model to address the relationship *in vivo* between human NAT1/mouse Nat2 and folate metabolism, and describe phenotypic effects of deleting the mouse *Nat2* gene in mice.

## Methods

### *Nat2* transgenic mouse maintenance and breeding

All work involving animals was carried out according to the UK Animals (Scientific Procedures) Act of 1986 under license from the UK Home Office.

The generation of a stable *Nat2* knockout line of mice by targeted insertion of a *lacZ*-containing cassette, has been described [11]. The *Nat2^∗null^* allele was bred from a129/Ola background onto two different genetic backgrounds (A/J and C57Bl/6, supplied by Harlan, UK) by backcrossing over ten generations. *Nat2*^−/−^, *Nat2*^+/−^ and *Nat2*^+/+^ animals used for analysis were generated by intercrossing, (mating *Nat2*^+/−^ male and *Nat2*^+/−^ females). On weaning, offspring were sexed on the basis of the external morphology of the genitalia. Ear biopsies were taken for genotyping and sexing and DNA isolated using Sigma GenElute mammalian genomic DNA miniprep kit. Offspring sex was confirmed by screening for the Sry gene using Sry and MyoG primers and PCR conditions detailed in [12]. Primers used for *Nat2* genotyping were as follows: Neo-T (forward, 5′CATCGCCTTCTATCGCCTTCT3′) and mNat2-910 (reverse, 5′TTCCAAGTACATGGAAGGACACC3′) were used to detect the *Nat2^∗null^* allele, with mNat2-1 (forward) 5′ATGGACATCGAAGCGTACTTTG3′, and mNat2-910 (reverse) used to detect the wild-type *Nat2* allele as described [11]. Genotypic and phenotypic data pertaining to each animal were stored and analyzed using Microsoft Access databases.

Adult (8- to 10-week-old) male C57Bl/6 *Nat2*^+/+^ and *Nat2*^−/−^ mice (*n* = 8 animals per genotype) were used for urine analysis in two independent experiments. All mice were fed on standard mouse chow given *ad libitum*. Animals given dietary folate supplement were fed *ad libitum* on mash made up with folic acid (20 μM, pH adjusted to pH 7.0) added to standard mouse chow at 20 mg folic acid/kg dry chow [13] over four to five days. Urine samples were collected during routine cage transfer on days 3–5 and samples from four animals per genotype were pooled for analysis.

To obtain embryos, timed matings were established, with noon on the day of the vaginal plug designated as embryonic day 0.5 (e0.5). Pregnant dams were killed by cervical dislocation. Embryos were dissected from the uterus into ice-cold 10 mM Potassium phosphate pH 7.5, 145 mM NaCl (phosphate buffered saline) containing 4% paraformaldehyde.

### Mass spectroscopy

*Mouse urine analysis by mass spectroscopy.* Urine samples (∼200 μl) were diluted 1:1 with ammonium hydroxide solution (40 μl of concentrated ammonium hydroxide in 1 ml water, pH 10) and centrifuged at 8000*g* for 10 min. The supernatant (∼400 μl) was applied to an anion-exchange cartridge (Oasis MAX, Waters, UK) previously washed with 1 ml of methanol and conditioned with 1 ml of water prior to sample application. Analytes were eluted from the cartridge with 5% ammonium hydroxide in water, followed by 1 ml of methanol and finally 1 ml of 5% formic acid in methanol.

All fractions were analysed by both negative- and positive-ion electrospray (ES) mass spectrometry (MS) and tandem mass spectrometry (MS/MS) on a Q-TOF Global (Waters, UK) instrument. The instrument was operated in the “V-mode” at a resolution of ∼7500 (FWHM). MS/MS spectra were recorded on selected precursor ions using a collision-energy of 20 eV.

## Results and discussion

### Metabolic effect of deleting mouse *Nat2*

**Fig. 1:**
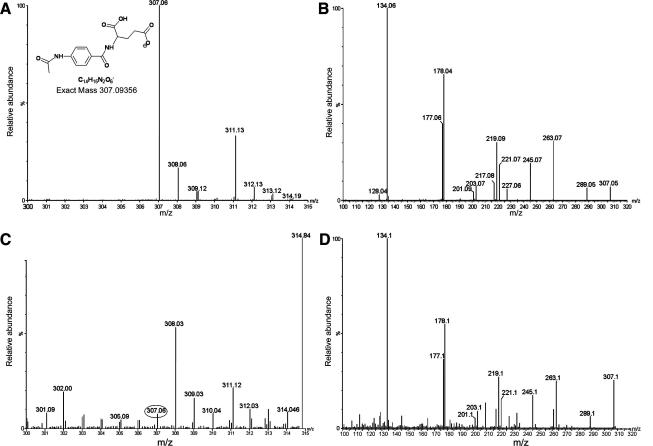
ES-MS and -MS/MS analysis of authentic acetyl-*p*ABAglu. (A) Negative ion ES-MS spectrum of authentic acetyl-*p*ABAglu, giving a [M-H]^−^ ion of *m*/*z* 307.06. Inset; chemical structure of the [M-H]^−^ ion of acetyl-*p*ABAglu showing exact mass. (B) MS/MS of the ion of [M-H]^−^ ion (*m*/*z* 307) from authentic acetyl-*p*ABAglu. (C) Negative ion ES-MS spectrum of urine from *Nat2*^+/+^ mice given dietary folate supplement. (D) MS/MS of the ion of [M-H]^−^ ion (*m*/*z* 307) from urine of *Nat2*^+/+^ mice given dietary folate supplement. See Table 1 for interpretation of fragment ions.

The ability of adult *Nat2^+/+^* and *Nat2^−/−^* mice to acetylate the folate catabolite *para*-aminobenzoylglutamate (*p*ABAglu) was tested by mass spectroscopic analysis of urine samples. Negative-ion ES-MS analysis of purified acetylated *p*ABAglu gave a [M-H]^−^ ion at *m*/*z* 307.06 (theoretical *m*/*z* 307.09). This compound eluted in the formic acid fraction from the anion-exchange column. The ion of *m*/*z* 307.06 was also found in ES-MS spectra of the formic acid anion-exchange fraction from the urine of *Nat2^+/+^* mice given a dietary folate supplement, indicating that acetyl-*p*ABAglu is present in their urine (Fig. 1). However, this ion was absent from the ES-MS spectra of all anion-exchange fractions from *Nat2*^−/−^ mouse urine. To confirm the identity of components having a mass of 307.06 in *Nat2^+/+^* mouse urine, the ion at *m*/*z* 307 found in the formic acid fraction from the anion-exchange column was further analysed by MS/MS, and the resultant spectrum compared to that of the [M-H]^−^ ion of purified acetyl-*p*ABAglu (Fig. 1, Table 1). The MS/MS spectra obtained from the ion of *m*/*z* 307 in urine of the *Nat2^+/+^* mice were essentially identical to that of standard acetyl-*p*ABAglu, however the corresponding spectra from *Nat2^−/−^* mice urine did not correspond to the acetyl-*p*ABAglu standard (Table 1). These data indicate that acetyl-*p*ABAglu is present in the urine of *Nat2^+/+^* mice given dietary folate supplements, but undetectable in urine from *Nat2^−/−^* mice.

Early work on human NAT1 enzyme demonstrated that the acetylation of NAT1-specific substrate *para*-aminobenzoic acid by NAT1 in human erythrocyte cytosols is inhibited *in vitro* by folic acid, and folate levels in these cells appears to show an inverse correlation with acetylation activity [14]. Subsequently, recombinant human NAT1 enzyme was shown to acetylate the folate catabolite *p*ABAglu [9,10]. The role of folate in cancer biology is complex, with folate supplements used to prevent tumour formation in normal tissues, although these supplements may accelerate the growth of established tumours, and antifolate agents have some efficacy as cancer treatments [15,16]. This duality shown by folate mirrors the effects of human NAT1 on cell growth. High level of expression of human NAT1 has been shown to enhance cell growth under certain conditions [17], but is associated with improved clinical outcome in ER positive breast cancer [8].

### *Nat2* deletion affects offspring sex ratios in A/J strain

**Supplementary Figure S1:**
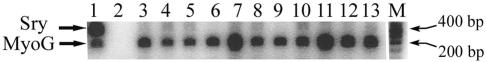
PCR screen for *Sry* gene in female offspring derived from intercross matings. DNA prepared from ear biopsies taken from animals identified as female from the appearance of external genitalia, was tested for the presence of the *Sry* gene using PCR. Lane 1: positive control, biopsy taken from a male, giving upper (441 bp) *Sry* product and lower (245 bp) *MyoG* bands. Lane 2: negative control, no DNA. Lanes 3–13 test samples identified as phenotypically female, lacking *Sry* product, with the presence of control *MyoG* product in each sample confirming DNA quality. Lane M, “1 kb plus” molecular weight marker.

*Nat2*^−/−^ mice are described as overtly aphenotypic [11]. However, on an A/J genetic background, within homozygous null litters there is a gender bias with a 1.5 fold excess of males (89 males; 59 females) that differs significantly from the predicted 1:1 ratio (*p* = 0.01) suggesting that homozygosity of the *Nat2^null^* allele may be deleterious to females, or beneficial in males. A reversed gender bias is seen in offspring from intercross matings (*Nat2^+/−^* M × *Nat2^+/−^* F), in which both parents are heterozygous (Table 2), with a ratio of males to females of 0.7:1 (184 males to 254 females *p* = 0.001). In these intercross litters, the female excess is made up of *Nat2*^+/+^ and *Nat2*^+/−^ animals (Table 2), again suggesting that in females, the wild-type allele has selective advantage. Together these results indicate that mutations at the *Nat2* locus have a gender-dependent effect on offspring survival. By screening for the *Sry* gene [12], we have confirmed that the skewed sex ratios do not result from sex reversal. (Supplementary Figure S1).

### Effect of *Nat2* deletion on neural tube closure

**Fig. 2:**
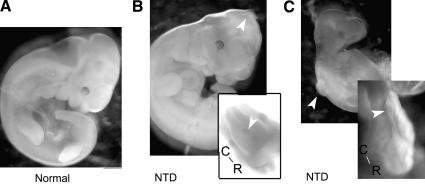
Wholemount views of embryos showing neural tube defects. Embryos derived by crossing C57Bl/6 *Nat2^+/+^* and *Nat2^−/−^* mice, and harvested at e10.5–e11.5. (A) Normal embryo (e11.5) viewed from right hand side. (B) Embryo (e11.5) with neural tube defect, showing incomplete closure of the anterior neuropore, viewed from right hand side in main panel; inset, dorsal view. (C) Embryo (e10.5) with neural tube defect at cervical level, viewed from right in main panel; inset, dorsal view. Arrowheads point to regions of incomplete closure of the neural tube. The orientation of the embryos is indicated in dorsal views; C, caudal; R, rostral.

We have tested whether *Nat2* genotype influences susceptibility to neural tube defects, using the C57Bl/6 strain, which is not prone to neural tube defects, and scoring embryos for neural tube defects at e10.5 and e11.5. Amongst Nat2^−/−^ offspring derived by incrossing, out of 64 embryos examined, only one embryo was observed to have a neural tube defect. To investigate the effects of an imbalance in parental *Nat2* phenotype, crosses were set up using *Nat2*^+/+^ and *Nat2*^−/−^ parents, generating *Nat2*^+/−^ offspring. The incidence of neural tube defects amongst *Nat2*^+/−^ offspring derived in this way was 14% (4/28). In three out of the four cases described in here, associated with the *Nat2 null* allele, the position of the defect is caudal to the hindbrain, at the cervical level (Fig. 2).

An allelic association between the orthologous human *NAT1* and orofacial clefting has been recorded [18] and a link between *NAT1* imbalance and developmental defects has been described for the human *NAT1* alleles with respect to the incidence of spina bifida [19]. In this human study, natural variants with little acetylating activity were found to have a protective effect amongst offspring, and both maternal and offspring genotypes affected the outcome. Gender bias towards females amongst exencephalic embryos both in mice and in men is well documented, for review [20]. The A/J strain is known to be susceptible to neural tube defects, and it is possible that the male bias seen amongst A/J *Nat2^−/−^* offspring is due to developmental abnormalities and lower survival rates amongst female embryos, although the numbers presented here are too small at present for statistical analysis.

Folate supplements have been used widely to prevent neural tube defects and to reduce the risk of colon cancer, reviewed in [15]. The data presented here indicate that deleting mouse *Nat*2 reduces the acetylation of the folate catabolite *p*ABAglu. Our results suggest that the complex relationship between human *NAT1* polymorphism and epithelial cancers [7] including breast cancer [8,17], and the influence of *NAT1* genotype on neural tube defects [19] may result from a functional link *in vivo* between NAT1 acetylation activity and folate metabolism.

## Figures and Tables

**Table 1:** MS/MS analysis of the ion of *m/z* 307 in the formic acid fraction from the anion-exchange column from urine of *Nat2*^+/+^ and *Nat2*^−/−^ mice

MS/MS Fragment ion *m*/*z*	Proposed fragment ion elemental formula	Authentic acetyl-pABAglu	Urinary 307.06 peak^a^		Acetyl-pABAGlu peak intensities^b^	Nat2^+/+^ peak intensities^b^
			Nat2^+/+^	Nat2^−/−^		
134.06	C_8_H_8_NO	*+*	*+*	−	1.0	1.0
178.04	C_9_H_8_NO_3_	*+*	*+*	−	0.66	0.65
219.09	C_12_H_15_N_2_O_2_	*+*	*+*	−	0.29	0.25
245.07	C_13_H_13_N_2_O_3_	*+*	*+*	−	0.18	0.18
263.07	C_13_H_15_N_2_O_4_	+	+	−	0.30	0.25

a Urine samples collected from adult male mice given a dietary supplement of folic acid were fractionated by anion-exchange chromatography and ions of *m*/*z* 307 further analysed by MS/MS.

b For both authentic acetyl-*p*ABAglu and *Nat2*^+/+^ derived samples, peak intensities are expressed relative to the peak of mass 134.06. +, peak present; −, peak absent. Experimental *m*/*z* values agree (<±0.03 Da) with theoretical *m*/*z* values given in the Table.

**Table 2:** Analysis of sex and genotype in A/J intercross offspring

*Nat2* genotype	Offspring numbers		
	Males	(Predicted)	Females
+/+	50	(55)	77
+/−	77	(110)	122
−/−	62	(55)	55
*χ*^2^	*p* < 0.01^a^		*p* < 0.01^a^

Inheritance of the *Nat2*^null^ allele was determined in offspring of intercross mating using PCR. Numbers of offspring predicted assuming independent segregation and Mendelian inheritance of *Nat2* (located on chromosome 8) and *Sry* gene (located on Y chromosome) are given in parentheses.

a Significant deviation from predicted values.
